# MRI-Monitored Intra-Tumoral Injection of Iron-Oxide Labeled *Clostridium novyi-NT* Anaerobes in Pancreatic Carcinoma Mouse Model

**DOI:** 10.1371/journal.pone.0116204

**Published:** 2014-12-30

**Authors:** Linfeng Zheng, Zhuoli Zhang, Khashayarsha Khazaie, Saurabh Saha, Robert J. Lewandowski, Guixiang Zhang, Andrew C. Larson

**Affiliations:** 1 Department of Radiology, First People’s Hospital, Shanghai Jiaotong University, Shanghai, China; 2 Department of Radiology, Northwestern University Feinberg School of Medicine, Chicago, Illinois, United States of America; 3 Robert H. Lurie Comprehensive Cancer Center, Chicago, Illinois, United States of America; 4 Department of Immunology, Mayo Clinic College of Medicine, Mayo Clinic, Rochester, Minnesota, United States of America; 5 BioMed Valley Discoveries, Kansas City, Missouri, United States of America; Brandeis University, United States of America

## Abstract

**Objectives:**

To validate the feasibility of labeling *Clostridium novyi-NT* (*C.novyi-NT*) anaerobes with iron-oxide nanoparticles for magnetic resonance imaging (MRI) and demonstrate the potential to use MRI to visualize intra-tumoral delivery of these iron-oxide labeled *C.novyi-NT* during percutaneous injection procedures.

**Materials and Methods:**

All studies were approved by IACUC. *C.novyi-NT* were labeled with hybrid iron-oxide Texas red nanoparticles. Growth of labeled and control samples were evaluated with optical density. Labeling was confirmed with confocal fluorescence and transmission electron microscopy (TEM). MRI were performed using a 7 Tesla scanner with T2*-weighted (T2*W) sequence. Contrast-to-noise ratio (CNR) measurements were performed for phantoms and signal-to-noise ratio (SNR) measurements performed in C57BL/6 mice (n = 12) with Panc02 xenografts before and after percutaneous injection of iron-oxide labeled *C.novyi-NT.* MRI was repeated 3 and 7 days post-injection. Hematoxylin-eosin (HE), Prussian blue and Gram staining of tumor specimens were performed for confirmation of intra-tumoral delivery.

**Results:**

Iron-oxide labeling had no influence upon *C.novyi-NT* growth. The signal intensity (SI) within T2*W images was significantly decreased for iron-oxide labeled *C.novyi-NT* phantoms compared to unlabeled controls. Under confocal fluorescence microscopy, the iron-oxide labeled *C.novyi-NT* exhibited a uniform red fluorescence consistent with observed regions of DAPI staining and overall labeling efficiency was 100% (all DAPI stained *C.novyi-NT* exhibited red fluorescence). Within TEM images, a large number iron granules were observed within the iron-oxide labeled *C.novyi-NT*; these were not observed within unlabeled controls. Intra-procedural MRI measurements permitted *in* *vivo* visualization of the intra-tumoral distribution of iron-oxide labeled *C.novyi-NT* following percutaneous injection (depicted as punctate regions of SI reductions within T2*-weighted images); tumor SNR decreased significantly following intra-tumoral injection of *C.novyi-NT* (p<0.05); these SNR reductions were maintained at 3 and 7 day follow-up intervals. Prussian blue and Gram staining confirmed presence of the iron-oxide labeled anaerobes.

**Conclusions:**

*C.novyi-NT* can be labeled with iron-oxide nanoparticles for MRI visualization of intra-tumoral deposition following percutaneous injection during bacteriolytic therapy.

## Introduction

The potential role of anaerobic bacteria as anti-cancer agents has been recognized for over one hundred years [1]–[8]. These bacteria, including *Salmonella typhimurium* (*S.typhimurium*), *Streptococcus pyogenes* (*S.pyogenes*), and *Clostridium novyi* (*C.novyi-NT non-toxic clone variant*) can grow in hypoxic tumor regions and lyse malignant cells by secreting lipases, proteases and other hydrolytic enzymes, and recruiting inflammatory cells to tumors eliciting anti-tumor immune responses. Bacteriolytic strategies offer unique advantages to combat a broad range of cancers often refractive to conventional chemotherapies and/or radiotherapies [1]–[9]. Recent studies have clearly demonstrated that *C.novyi-NT* (administered either systemically or intra-tumorally with percutaneous injection) can elicit marked regression of tumors in pre-clinical research settings [3], [5], [8], [9]. Most recently the efficacy of *C.novyi-NT* has been evaluated in companion dog models and current human trials are underway to demonstrate therapeutic efficacy in clinical settings (NCT01118819 and NCT01924689) [10]–[13]. While the optimal administration route remains unclear, intra-tumoral injections may be warranted to avoid potential complications arising from systemic administration.

The efficacy of bacteriolytic therapy is strongly influenced by the biodistribution of the administered anaerobes. During percutaneous injection procedures, *in* *vivo* depiction of a) the targeted tumor tissues, b) infusion needle position, and c) the delivered aneorobes (post-infusion) may be critical for intra-procedural optimization and/or early prediction of longitudinal outcomes. Magnetic resonance imaging (MRI) should be ideal for this role offering many potential advantages including excellent soft tissue contrast, no requirement for radioactive isotopes, and none of the depth penetration issues associated with the bioluminence methods often used to image bacterial distributions in mouse models [11], [14]–[17].

Exogenous labeling with iron oxide agents has permitted MRI of cell migration and determination of resulting biodistributions [18]. Superparamagnetic iron oxide (SPIO) labeling has been widely used to visualize stem cell migration, homing of dendritic cell cancer vaccines to lymph nodes, and natural killer cell delivery to hepatocellular carcinoma [16], [17], [19]–[22]. SPIO nanoparticles are sequestered within cells, thus inducing field inhomogeneities due to magnetic susceptibility differences [23], [24]. These field perturbations lead to signal reductions thus permitting T2- and/or T2*-weighted MRI of SPIO-labeled cell delivery and migration. Currently the most widely used approach for magnetic labeling of eukaryotic cells involves co-culture with the SPIO probes for cellular endocytosis, similar co-culturing processes should be feasible for labeling bacteria [16], [19], [25], [26].

The purpose of our current study was to a) validate the feasibility of labeling *C.novyi-NT* anaerobes with commercially available iron-oxide nanoparticles for MRI and b) demonstrate the potential to use MRI to visualize intra-tumoral delivery of these iron-oxide labeled *C.novyi-NT* during percutaneous injection procedures in a pancreatic carcinoma mouse model.

## Materials and Methods

The present study was carried out in strict accordance with the guidelines from the National Institutes of Health for the Care and Use of Laboratory Animals. The use of mice was approved by Institutional Animal Care and Use Committee (IACUC) of Northwestern University.

### *C.novyi-NT* Culture

*C.novyi-NT* spores were provided by BioMed Valley Discoveries (Kansas City, MO). The spores were cultured in Reinforced medium for *Clostridia* (Himedia Laboratories Pvt. Ltd, India) for one week to generate the vegetative form of *C.novyi-NT* using a BD Gaspak^TM^ EZ Anaerobe Pouch System (Sparks, MD, USA) at 37°C in an incubator (Thermo Scientific, Germany). The vegetative form of the *C.novyi-NT* continued to be cultivated on Reinforced medium in same setting. The *C.novyi-NT* population in each culture was estimated by measuring the optical absorbance at a wavelength of 600 nanometers (OD_600_) using SpectraMax M5 and SoftMax PRO Software, and *C.novyi-NT* concentration calculated using the formula 1 OD_600_ = 1×10^9^ cells/ml [27]. All subsequent experiments with each culture were performed after achieving an OD_600_ of 0.2–0.4.

### *C.novyi-NT* Labeling and Growth Curve Assay

*C.novyi-NT* suspensions were centrifuged at 1250 rpm for 10 min. After discarding supernatant, pellets of *C.novyi-NT* were resuspended in 5 ml fresh medium supplemented with final amount of 40 pg Fe/*C.novyi-NT* NIMT FeOlabel Texas Red iron-oxide particles (monodispersed spherical shaped nanoparticles, 11±1 nm core size, approximately 45±5 nm hydrodynamic diameter; Genovis AB, Lund, Sweden) and incubated at 37°C for labeling period of 48 hours. At 2, 4, 6, 8, 12, 24, 36 and 48 hours of exposure to the iron-oxide labeling material, 100 µl of *C.novyi-NT* suspension were removed for assessment of growth rates according to OD_600_ measurements. Identical procedures were then performed without exposure of *C.novyi-NT* to iron-oxide labeling materials with these latter growth rates by OD_600_ measurements serving as controls. Each of these studies was performed in triplicate independently.

For fluorescence microscopy, the iron-oxide labeled *C.novyi-NT* (24 hr labeling period) and unlabeled *C.novyi-NT* were washed with phosphate buffered saline (PBS) twice for 1 min and then fixed with 10% neutral formalin for 10 min. The formalin was next removed and specimens washed with PBS. The iron-oxide labeled *C.novyi-NT* were mixed with Vortex Mixer to dilute 10-fold. Then 10 µl of dilution suspension were smeared upon a glass slide. After drying, 10 µl of 4′,6 diamidino-2-phenylindole (DAPI; Vector Laboratories, Inc, Burlingame, CA) was added to stain each slide for 5 min. Finally, the slides were sealed with a coverslip. Fluorescence images were captured under a 100× oil immersion lens with Nikon A1R laser scanning confocal microscope (Nikon Instruments Inc., Japan). Within co-registered images, double stained bacteria (exhibiting both Texas Red and DAPI) were defined as those having been successfully labeled with the iron-oxide agent. The labeling efficiency (% of successfully iron-oxide labeled *C.novyi-NT*) was measured within five randomly selected fields for each sample in three independent experiments.

For transmission electron microscopy (TEM) of roughly 3.4×10^8^ iron-oxide labeled *C.novyi-NT* (labeling 24 hrs) and unlabeled *C.novyi-NT* were collected and fixed using 2.5% glutaraldehyde fixative solution. After routine TEM processing and sectioning, the samples were observed using an FEI Tecnai Spirit G2 120 kV TEM (FEI company, Hillsboro, OR) system to confirm uptake of the iron-oxide labeling material.

### Panc02 Cell Culture and Mouse Xenograft Tumor Model

The pancreatic ductal adenocarcinoma cell line Panc02 was obtained from National Cancer Institute (Frederick, MD) and cultured in RPMI 1640 medium supplemented with 10% fetal bovine serum (FBS), 2 mmol/l glutamine, 1 mmol/l pyruvate, 100 IU/ml penicillin and streptomycin respectively in a humidified incubator containing 5% CO_2_ at 37°C. Cell viability was tested by Trypan blue staining and a cell viability >90% was considered suitable for the following tumor inoculation methods.

Tumor implantation was performed according to previously described procedures [15], [17]. 2×10^6^ early-passage Panc02 cells were harvested and re-suspended in 100 µl of PBS. These Panc02 cell were then implanted subcutaneously into the bilateral flanks of female C57BL/6 mice (4 weeks old, range from 15–20 gram weight; Charles River, Wilmington, MA). These mice were provided a standard laboratory diet, free access to water and normal light/dark cycle. All efforts were made to minimize suffering for the mice. Tumors were allowed to grow for 1–2 weeks after implantation prior to imaging and bacteria injection procedures. The longest length and maximum width were measured using a caliper, and the tumor sizes were calculated using the tumor size formula = longest length×maximum width [17].

### MRI

A Bruker 7 Tesla ClinScan MRI horizontal bore scanner (Bruker, Billerica, MA) with dedicated mouse surface coil was employed for all phantom and mouse imaging studies.

### Phantom Studies

Separate suspensions with samples of 5.1×10^8^ iron-oxide labeled *C.novyi-NT* and 5.1×10^8^ unlabeled *C.novyi-NT* were washed with PBS (1 min, two times) to remove any residual iron particles and centrifuged (1250 rpm, ×5 min) horizontally in 1.5 ml Eppendorf tubes. Then the PBS was removed and replaced with 2.8% agarose solution. Finally, each Eppendorf tube was inserted in a plastic test tube after filling with 2.8% agarose solution before placing in a refrigerator to solidify. T2*-weighted (T2*W) images were obtained using the following sequence and parameters: Gradient echo (GRE) sequence, 35×35 mm^2^ field-of-view (FOV), 256×256 matrix, 20° flip-angle (FA), 300 Hz/pixel bandwidth, 0.4 mm slice thickness, Repetition time (TR)/echo time (TE) = 100/10 ms, 2 signal averages, 0.14×0.14×0.40 mm^3^ voxel size.

### *In vivo* Studies

Before and during MRI, animals were anesthetized with a mixture of isoflurane and oxygen (Isoflurane Vaporizer, Vaporizer Sales and Services, Rockmart, GA). Mouse temperature was monitored continuously and controlled with a water bed. Heart rate, respiration rate, and blood pressure were monitored with an MRI-compatible small animal gating system (SA Instruments, Stony Brook, NY).

T1-weighted (T1W), T2W and T2*W images were acquired prior to iron-oxide labeled bacteria injection using turbo spin echo (TSE) and gradient echo (GRE) sequences with specific acquisition parameters described in Table 1. Baseline MRI measurements were performed prior to percutaneous placement of a catheter (26 ga×¾ in 19 mm; Monoject Veterinary Catheter; Tyco healthcare Group Lp, Mansfield, MA) with iterative follow-up imaging to permit adjustment to the position of catheter tip prior to removal of stylus for gentle manual infusion of the iron-oxide labeled bacteria from 1 ml syringe (suspension of 3.4×10^7^ *C.novyi–NT* within PBS, total volume 0.1 ml); after infusion both catheter and syringe held in place for 2 min prior to removal. Post-injection T2*W images were acquired immediately after injection procedure as well as 3 days and 7 days after the injection procedure.

**Table 1:** Parameters for T1W, T2W and T2*W Sequences.

	Sequence	TR (ms)	TE (ms)	FOV (mm^2^)	Matrix	FA	Slice thickness (mm)	Bandwidth	Signal averages	Voxel-size (mm^3^)
T1W	GRE	20	3.6	35×35	256×256	20°	0.50	300 Hz/pixel	8	0.14×0.14×0.50
T2W	TSE	3000	40	35×35	256×256	90°	0.50	300 Hz/pixel	8	0.14×0.14×0.50
T2*W	GRE	100	10	35×35	35×35	20°	0.40	300 Hz/pixel	8	0.14×0.14×0.40

Note: T1W = T1-weighted, T2W = T2-weighted, T2*W = T2*-weighted, GRE = Gradient echo, TSE = Turbo spine echo, TR = Repetition time, TE = Echo time, FOV = Field of view, FA = Flip angle.

### Histology

The mice were euthanized immediately with carbon dioxide after final MRI exams. Next, each tumor was harvested and fixed with 10% neutral formalin solution. The tumor samples were submitted to a Mouse Histology and Phenotyping Laboratory (MHPL) core for hematoxylin-eosin (HE) staining, Prussian blue staining, and Gram staining. Resulting histology slices were scanned at 20× magnification and digitized using the TissueFAXS system (TissueGnostics, Los Angeles, CA).

### Data and Statistical Analysis

The relative contrast-to-noise (CNR) was measured within phantom images. Separate region of interest (ROI) were drawn to measure the mean signal intensity (SI) within *C.novyi-NT* phantoms (*SI_C.novyi-NT_*) and adjacent agarose (*SI_agar_*) and noise estimated as the standard deviation (SD) of SI within region external to the phantom (σ*_n_*). CNR was calculated as follows: CNR = (*SI_C.novyi-NT_−SI_agar_*)/σ*_n_* [28].

Quantitative analysis of *in* *vivo* MRI images acquired before and after iron-oxide labeled *C.novyi-NT* injection were performed by measuring the signal-to-noise ratio (SNR) of tumor tissues relative to skeletal muscle. Tumor ROI were drawn to encompass all tumor tissue within slice bisecting center of the tumor to measure mean tumor SI (*SI_tumor_*). A separate ROI was then drawn to measure the SD of SI within psoas major muscle (σ*_muscle_*). The SNR was calculated as follows: SNR =  *SI_tumor_*/σ*_muscle_* [29].

Data was expressed as mean±SD. For comparison of bacterial proliferation rates, the OD_600_ for unlabeled and iron-oxide labeled *C.novyi-NT* at time point from three independent experiments, the two-tailed Paried-samples *t* test was carried out. For comparison of CNR changes in phantoms and SNR changes *in* *vivo* studies, a one-way analysis of variance (ANOVA) followed with *LSD* multiple comparison tests were employed; differences with p<0.05 were considered statistically significant. All statistical analyses were performed using a statistics software program (SPSS Statistics, IBM, Armonk, NY).

## Results

### *C.novyi-NT* Iron-oxide Labeling and Validation

**Figure 1:**
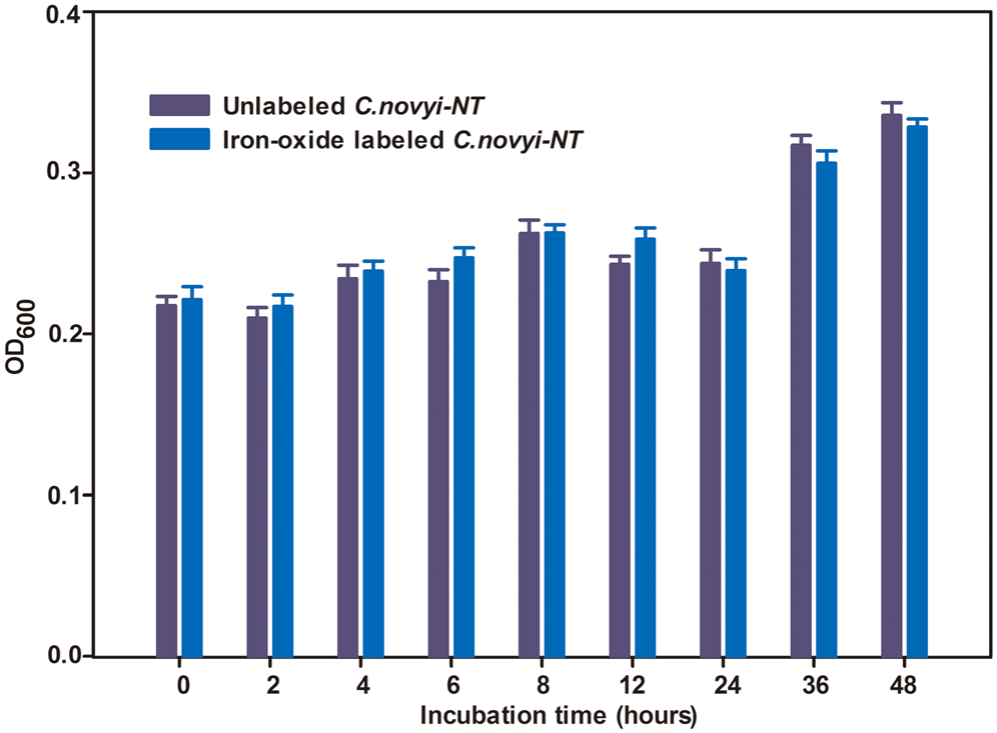
OD_600_ of *C.novyi-NT* at different time points after iron-oxide labeling. There was no significance between iron-oxide labeled and unlabeled *C.novyi-NT* OD_600_ measurements (p>0.05 for comparisons at each time point, n = 3).

**Figure 2:**
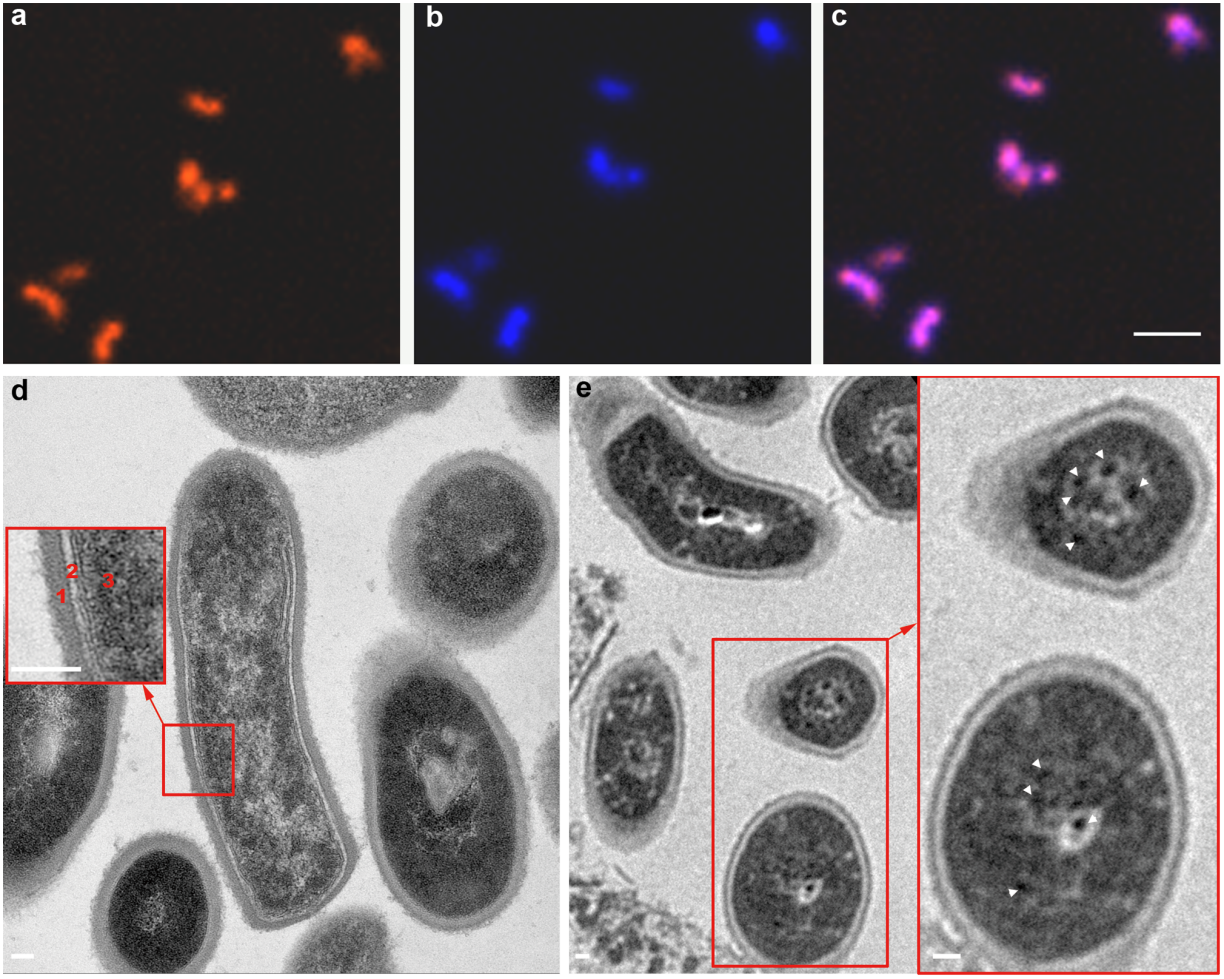
Representative confocal fluorescence microscopy and TEM images of Texas Red iron-oxide labeled *C.novyi-NT*. Texas Red particles were observed within the *C.novyi-NT* (a). *C.novyi-NT* were stained blue with DAPI (b). Merged image (c) demonstrates the co-registration of Texas Red and DAPI staining. TEM of control unlabeled *C.novyi-NT* (d) with inset depicting the bacterial wall (1), light plasma membrane (2), and relatively homogeneous dark-stained cytoplasm and nucleoid core (3). Within TEM image of iron-oxide labeled *C.novyi-NT* (e), a large number of punctate dark-stained iron granules were observed within the bacteria (white arrowheads within inset). Scale bars: a, b, and c = 6 µm; d, e, and inset in d and e = 60 µm.

The growth curves for iron-oxide labeled *C.novyi-NT* were similar to that of unlabeled *C.novyi-NT* according to measured OD_600_ values. Iron-oxide labeling did not influence *C.novyi-NT* growth (p>0.05 for comparison of iron-oxide labeled *versus* unlabeled OD_600_ at each time-point) (Fig. 1). Under confocal fluorescence microscopy, the iron-oxide labeled *C.novyi-NT* exhibited a uniform red fluorescence consistent with observed regions of DAPI staining (Fig. 2a–c). Labeling efficiency was 100%, with all DAPI stained *C.novyi-NT* concurrently exhibiting red fluorescence. Representative TEM images of *C.novyi-NT* are shown in Figs. 2d and 2e. In control example, TEM images clearly exhibit the bacterial wall, light plasma membrane, and relatively homogeneous dark-stained cytoplasm and nucleoid core (Fig. 2d), similar to architectures described in prior literature [30]. Within iron-oxide labeled *C.novyi-NT*, a large number of punctate dark-stained iron granules were observed within the bacteria (Fig. 2e).

### *In vitro* Phantom Study

**Figure 3:**
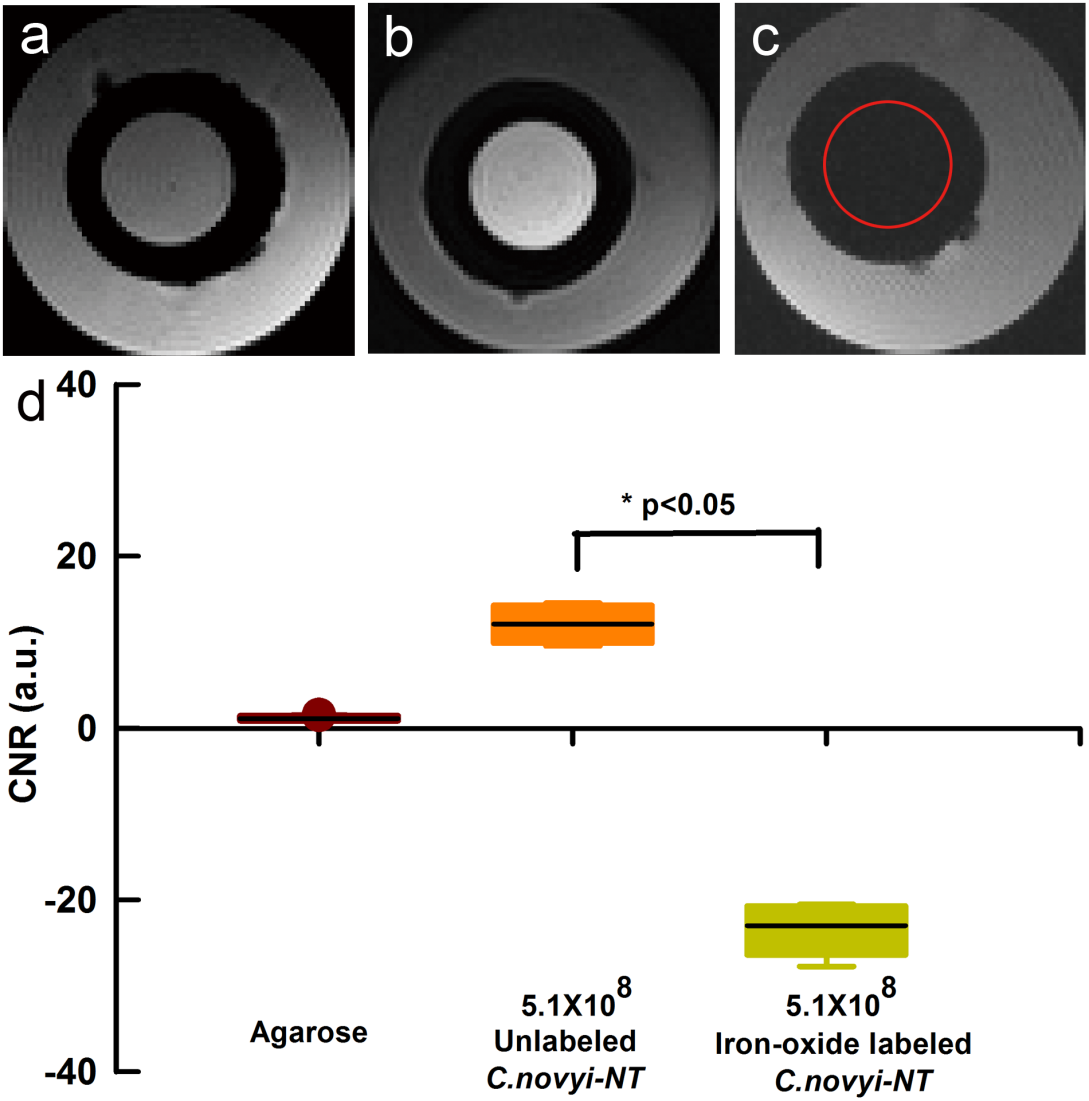
MRI of phantoms containing control unlabeled samples of *C.novyi-NT* and iron-oxide labeled *C.novyi-NT*. Axial T2*-weighted images of agarose (a), suspension of 5.1×10^8^ unlabeled *C.novyi-NT* (b), and 5.1×10^8^ iron-oxide labeled *C.novyi-NT* (c). CNR measurements from these phantom images are shown in (d). The red cycle within (c) indicates position of the iron-oxide labeled *C.novyi-NT* phantom vial.

The T2*W SI decreased significantly for those phantoms containing iron-oxide labeled *C.novyi-NT* compared to those phantoms with unlabeled controls (Figs. 3b and 3c). CNR (relative to adjacent agarose) dropped from 12.1±2.3 in control phantoms to −23.0±3.3 in the phantom containing the iron-oxide labeled *C.novyi-NT* (Fig. 3d; p<0.05 for latter comparisons).

### *In vivo* Study

A total of 24 Panc02 tumors were grown in 12 mice; growth of these tumors was clearly observed and verified within the acquired T2W anatomic images. Overall tumor size was 0.7±0.3 cm^2^ (mean±SD, n = 24). Of these 24 tumors, 6 tumors (size 0.8±0.4 cm^2^) demonstrated large areas of liquefactive necrosis (these central necrotic zones were >5 mm in diameter within the T2W images); the remaining tumors (size 0.6±0.3 cm^2^) did not exhibit these extensive regions of liquefactive necrosis.

**Figure 4:**
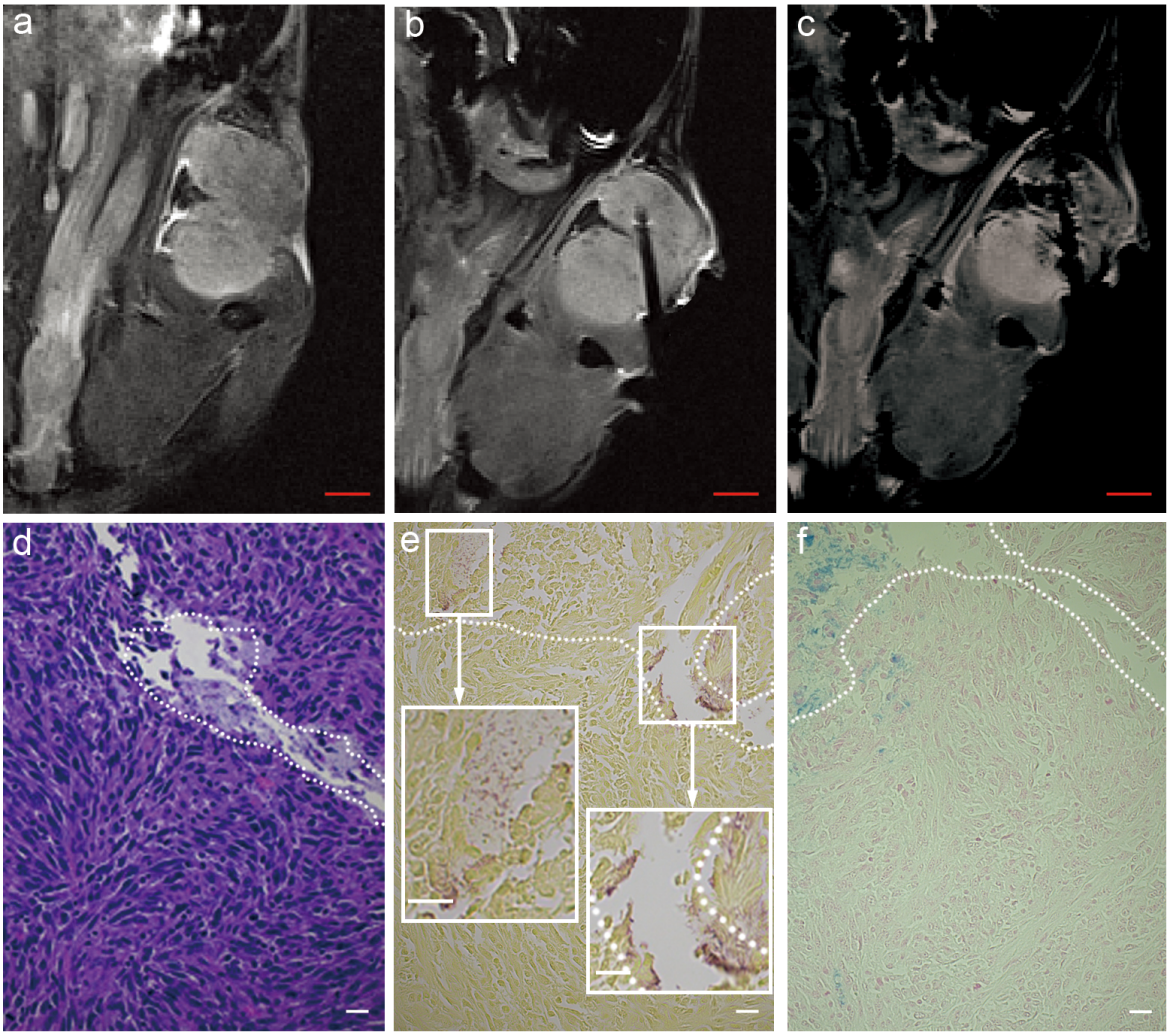
MRI-monitored injection of iron-oxide labeled *C.novyi-NT* into Panc02 tumors in mouse pancreatic cancer model. Coronal T2*-weighted image of tumor pre-injection and prior to injection catheter placement (a), after catheter placement within the tumor (b), and post-injection of iron-oxide labeled *C.novyi-NT* (c). Hematoxylin-eosin (HE) staining of tumor specimen (d) and both Gram staining and Prussian blue staining from adjacent sections shown in (e) and (f), respectively. The white dashed zones in (d-f) depict the catheter tract and distal zones of iron-oxide labeled anaerobe deposition. Size bar for MRI image a, b and c = 2 mm. Scale bar within d, e, f, insets of e = 20 µm.

Representative T2*W images, acquired in coronal orientation during MRI-monitored percutaneous intra-tumoral injection of iron-oxide labeled *C.novyi-NT,* are shown in Fig. 4a–c. The solid tumor in this example was hyperintense relative to adjacent skeletal muscle (Fig. 4a); injection catheter position was readily visualized as signal void within the T2*W image (Fig. 4b). Infusion of the iron-oxide labeled *C.novyi-NT* elicited signal reductions within multiple positions distal to the tip of the infusion catheter as well as along the edge of the infusion catheter (likely the result of reflux along the catheter placement track) (Fig. 4c). The catheter track was readily identified within H&E slides (Fig. 4d) with iron-oxide labeled *C.novyi-NT* deposition observed at positions both adjacent and distal to the tip of the infusion catheter track within Gram staining and Prussian blue slides (Figs. 4e and 4f).

**Figure 5:**
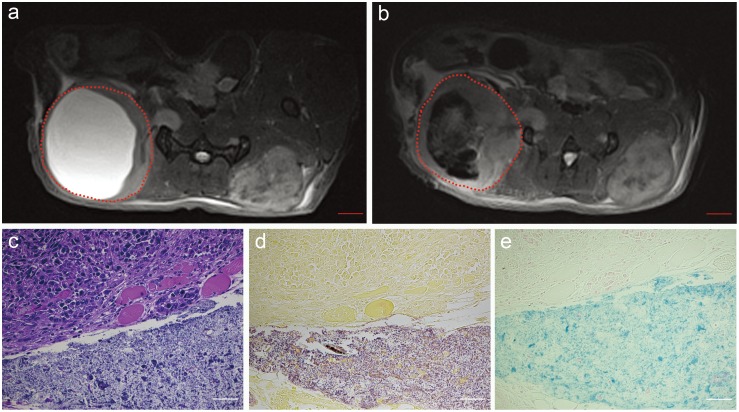
MRI measurements following percutaneous injection of iron-oxide labeled *C.novyi-NT* into a Panc02 tumor with an expansive central zone of liquefactive necrosis. Axial T2*-weighted images acquired pre-injection (a) and immediately post-injection (b). The red dashed zones in (a) and (b) indicate the tumor position. Hematoxylin-eosin (HE) staining (c), Gram staining (d) and Prussian blue staining (e) from tissue sample at border between solid viable tumor and necrotic region depict the broad distribution of the iron-oxide labeled anaerobes with the necrotic zones. Size bar for MRI image a and b = 2 mm. Scale bars within c, d and e = 50 µm.

Representative axial T2*W images acquired before and after percutaneous injection of iron-oxide labeled *C.novyi-NT* into tumor with an expansive central zone of liquefactive necrosis (injection catheter tip was positioned at the center of this hyperintense cystic space) (Figs. 5a and 5b). T2*W images permitted immediate post-injection depiction of the intra-tumoral distribution of the iron-oxide labeled *C.novyi-NT* anaerobes (Fig. 5b). Histology slides clearly depict zones of viable tumor and liquefactive necrosis (H&E) (Fig. 5c), *C.novyi-NT* anaerobes within the necrotic tissue (Gram staining) (Fig. 5d), and corresponding presence of iron-oxide labels within the same zone (Prussian blue) (Fig. 5e).

**Figure 6:**
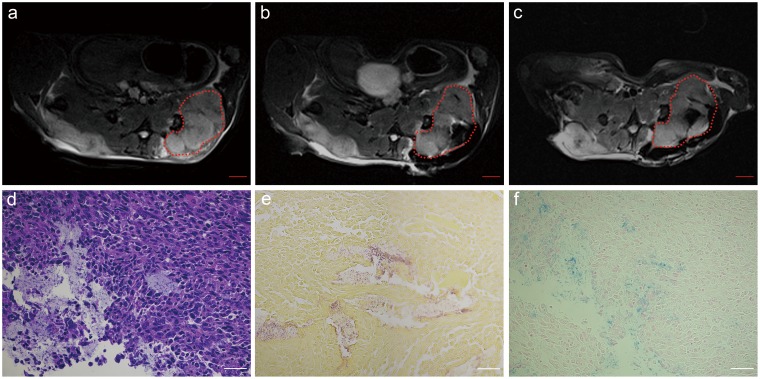
Serial MRI studies after MRI-monitored injection of iron-oxide labeled *C.novyi-NT* into a solid Panc02 tumor. Axial T2*-weighted acquired before (a), immediately after percutaneous injection (b), and 7-days after injection of iron-labeled *C.novyi-NT* (c) (areas within these dashed lines indicate tumor region). Hematoxylin-eosin (HE) staining (d), Gram staining (e) and Prussian blue staining (f) histology slide depicted zones of viable tumor and necrosis, *C.novyi-NT* anaerobes within necrotic areas, and corresponding presence of iron-oxide probe labels within these same zones. Size bar for MRI image a, b and c = 2 mm. Scale bars within d, e and f = 50 µm.

Axial T2*W images acquired before, immediately after percutaneous injection, and 7-days after injection of iron-oxide labeled *C.novyi-NT* into solid tumor are shown in Figs. 6a–c (injection catheter tip was positioned at tumor center). T2*W images permitted immediate post-injection depiction of the intra-tumoral delivery of the iron-oxide labeled *C.novyi-NT* as well as the accompanying retrograde leakage of the labeled anaerobes into subcutaneous spaces immediately adjacent to the tumor (Fig. 6b). 7-days after the initial injection, T2*W images continue to depict iron-oxide labeled *C.novyi-NT* within adjacent subcutaneous spaces and a redistribution of the anaerobes primarily to the central tumor core (Fig. 6c). Histology slides clearly depict zones of viable tumor and necrosis (H&E) (Fig. 6d), *C.novyi-NT* anaerobes within a necrotic area (Gram staining) (Fig. 6e), and corresponding presence of iron-oxide probe labels within the same zones (Prussian blue) (Fig. 6f).

**Figure 7:**
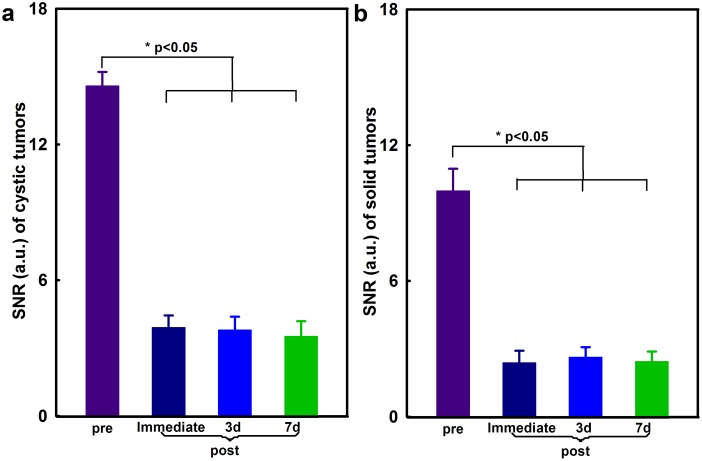
Tumor SNR measurements within T2*-weighted images before and after percutaneous injection of iron-oxide labeled *C.novyi-NT* into cystic Panc02 tumors with extensive zones of liquefactive necrosis (a, n = 6) and into solid Panc02 tumors (b, n = 6) with follow-up measurements performed immediately after injection and at 3-day and 7-day intervals post-injection. *p<0.05 for all pre- *vs* post-injection SNR measurements.

Tumors SNR decreased significantly following intra-tumoral injection of iron-oxide labeled *C.novyi-NT* (Figs. 7a and 7b). SNR measurements were 14.59±0.61 (pre-injection), 3.91±0.53 (immediately post-injection), 3.80±0.59 (3 days post-injection), and 3.52±0.66 (7 days post-injection) within cystic tumors (Fig. 7a) and 9.98±0.97 (pre-injection), 2.38±0.53 (immediately post-injection), 2.63±0.44 (3 days post-injection), and 2.44±0.44 (7 days post-injection) within solid tumors, respectively (Fig. 7b) (p<0.05 for all pre- *vs* post-injection SNR measurements, respectively).

## Discussion

Anaerobe colonization of tumor tissues is critical to the efficacy of bacteriolytic therapies. Percutaneous intra-tumoral injection permits targeted administration but both intra-procedural and/or post-procedural imaging of anaerobe delivery may be critical for patient-specific optimization of these administration procedures; imaging measurements may also permit early predictions on longitudinal outcomes based upon observed biodistribution of the administered anaerobes. These studies demonstrated that *C.novyi-NT* can be readily labeled with iron-oxide contrast materials to permit *in* *vivo* imaging following percutaneous intra-tumoral injection procedures. Iron-oxide labeling was highly efficient and had no observed impact upon *C.novyi-NT* growth rates. Both phantom and *in* *vivo* imaging studies demonstrated that the iron-oxide labeled *C.novyi-NT* produced strong T2*-weighted image contrast with the labeled anaerobes readily visible within targeted tumor tissues for extended periods post-injection. Given the anticipated importance of *C.novyi-NT* biodistributions to treatment outcomes, these non-invasive imaging methods are anticipated to offer a valuable means to optimize injection procedures and/or provide early predictions of response.

Given strong soft-tissue contrast characteristics, the lack of ionizing radiation, and translational potential, MRI is increasingly being advocated for a broad range of cellular and molecular imaging applications. Iron-oxide contrast materials have already been widely utilized for imaging exogenously labeled cells in both pre-clinical research studies and early translational clinical settings. Similar approaches are now being used for *in* *vivo* imaging of bacteria. Recently, magnetite-forming genes permitted *in* *vivo* imaging of magnetotactic AMB-1 bacteria for cancer visualization in pre-clinical xenograft tumor models [31] and bacterial ferritin-expressing *Escherichia coli* permitted *in* *vivo* MRI of tumor colonization [32]. Additional groups have recently developed methods to manipulate bacterial surface charges for attachment of iron-oxide particles for bacteria labeling and imaging in pre-clinical infection models [14]. In the current study, we demonstrated that co-culturing *C.novyi-NT* anaerobes with iron-oxide nanoparticle based contrast material permits uptake for labeling and subsequent *in* *vivo* imaging in the setting of bacteriolytic therapy.

The current study had several limitations. First, the animal model was limited to superficial, implanted tumors rather than spontaneously grown tumors as would be anticipated in clinical settings. Second, the current study used T2*-weighted imaging methods to visualize the iron-oxide labeled bacteria; additional studies are warranted to investigate the potential to use voxel-wise T2 and/or T2* mapping approaches for fully quantitative relaxometry measurements. Third, we provided only a qualitative validation of the iron-oxide labeling of the bacteria and did not perform quantitative studies of iron uptake using inductively coupled plasma/optical emission spectrometry (ICP-OES). Fourth, the focus of these studies was limited to labeling *C.novyi-NT* anaerobes and demonstrating the feasibility to visualize intra-tumoral delivery after percutaneous injection. Prior studies have already validated that *C.novyi-NT* anaerobes can induce marked regression of Panc02 tumors [5]; future studies will be valuable to compare imaging-based assessments of intra-tumoral *C.novyi-NT* delivery to outcomes in this pancreatic cancer animal model. Finally, while the Panc02 model was effective for the current study permitting relatively reproducible tumor growth within immunocompetent animals, genetically-engineered KPC mice [33], [34], providing a more clinically relevant model of pancreatic adenocarcinoma, may be more valuable for future studies of longitudinal therapeutic efficacy.

In summary, our results demonstrate that *C.novyi-NT* anaerobes can be labeled with iron-oxide nanoparticles for MRI visualization of intra-tumoral delivery. These methods offer the potential to serve as an important tool for intra-procedural monitoring and post-procedural follow-up during bacteriolytic therapy using *C.novyi-NT*.

## Supporting Information

S1 ARRIVE Checklist **The ARRIVE Guidelines Checklist.**
(DOC)
